# Affective and physiological responses to human body odors in social anxiety – a pilot study on the possible effects as catalyst for treatment

**DOI:** 10.1192/j.eurpsy.2023.240

**Published:** 2023-07-19

**Authors:** E. Vigna, C. Cecchetto, E. Dal Bò, G. Hadlaczky, D. Wasserman, V. Carli

**Affiliations:** 1 National Centre for Suicide Research and Prevention of Mental Ill-Health, Karolinska Institutet, Solna, Sweden; 2 Department of General Psychology, University of Padova, Padova, Italy

## Abstract

**Introduction:**

Understanding the way chemistry influences human communication is important since the reaction to chemosignals has many implications for science and society.

Numerous research points out that human beings are able to identify feelings of fear and happiness through smell. Such emotional information can lead to approach-avoidance behaviors or changes in affective state. Moreover, a heightened sensitivity to social odors has been shown in subjects with social anxiety symptoms. However, more detailed research on the connection between olfaction, affective psychiatric disorders and interpersonal social communication is required.

POTION is an EU funded project within the Horizon2020 initiative that aims at understanding the nature of chemosignals in humans and their sphere of influence on social interaction. Whitin this project, we conducted a preliminary study showing that individuals with social anxiety symptoms benefited from mindfulness training especially when exposed to social chemosignals. A significant reduction in anxiety symptoms was achieved with both the happiness (t(25)=4.37, p=0.029) and the fear (t(25)=4.35, p=0.031) chemosignals. Moreover, individuals exposed to the happiness chemosignal exhibit highier vagal tone compared to subjects exposed to fear chemosignals (p = 0.026), indicating overall increased well-being.

**Objectives:**

Given the exploratory nature of the preliminary study, an hypothesis driven pilot-RCT with larger sample size and refined design has been conducted. The aim was to further explore the catalyst effect of body odor on anxiety reduction. Notably, if the odor groups (happiness, fear or neutral) differ with the control group (clean air) and if they differ between each other in the outcome measure.

**Methods:**

To this end, 96 participants with social anxiety symptoms (women aged between 18 to 35) were randomly allocated to one exposure group (happiness, fear or neutral human body odor or clean air) and followed a mindfulness intervention while being exposed to the odor. Psychological outcomes were measured before and after the intervention through the State-Trait Anxiety Inventory. During the intervention participants’ skin conductance and heart rate was also measured.

Analysis of variance will be performed to assess psychological outcome differences between and within groups, as well as interactions (GroupxTime).

**Results:**

Results of the study will be available and presented at the time of the congress.

**Conclusions:**

This study represents an advancement in the field mental health as it explores the potential impact of using human chemosignals in the clinical setting.

**Disclosure of Interest:**

None Declared

